# Role of Sulfur Metabolism Gene and High-Sulfur Gene Expression in Wool Growth Regulation in the Cashmere Goat

**DOI:** 10.3389/fgene.2021.715526

**Published:** 2021-08-18

**Authors:** Yuan Chai, Yanyong Sun, Bin Liu, Lili Guo, Zaixia Liu, Le Zhou, Lingli Dai, Chunyan Jia, Wenguang Zhang, Chun Li

**Affiliations:** ^1^College of Animal Science, Inner Mongolia Agricultural University, Hohhot, China; ^2^College of Animal Science and Veterinary Medicine, Tianjin Agricultural University, Tianjin, China; ^3^Nei Mongol BioNew Technology Co., Ltd., Hohhot, China; ^4^Kunming Institute of Zoology, Chinese Academy of Sciences, Kunming, China; ^5^College of Animal Science and Technology, Inner Mongolia University for Nationalities, Tongliao, China

**Keywords:** high sulfur protein gene, sulfur metabolic gene, allele specific expression, melatonin, hair growth

## Abstract

Sulfur, an essential mineral element for animals, mainly exists in the form of organic sulfur-containing amino acids (SAAs), such as cystine, methionine, and cysteine, within the body. The content, form, and structure of sulfur play an important role in determining the wool fiber quality. In addition, keratin-associated proteins, one of the most crucial wool fiber components, are rich in SAAs. However, sulfur metabolism from the blood to the skin and hair follicles remains unclear. In this study, we analyzed high-sulfur protein gene and sulfur metabolism genes in the cashmere goat and explored the effects of melatonin on their expression. In total, 53 high-sulfur protein genes and 321 sulfur metabolism genes were identified. We found that high-sulfur protein genes were distributed in the 3–4 and 144M regions of chromosome 1 and the 40–41M region of chromosome 19 in goats. Moreover, all year round, allele-specific expression (ASE) is higher in the 40–41M region of chromosome 19 than in the other regions. Total of 47 high-sulfur protein genes showed interaction with transcription factors and cofactors with ASE. These transcription factors and cofactors were inhibited after melatonin implantation. The network analysis revealed that melatonin may activate the sulfur metabolism process via the regulation of the genes related to cell energy metabolism and cell cycle in the skin, which provided sufficient SAAs for wool and cashmere growth. In conclusion, our findings provide a new insight into wool growth regulation by sulfur metabolism genes and high-sulfur protein genes in cashmere goats.

## Introduction

The sulfur content directly affects the properties of wool fibers, including cashmere wool fibers. Notably, sulfur-containing amino acids (SAAs), differentially expressed genes (DEGs), methionine, and cysteine play an important metabolic and functional role in animals. In a study, SAA supplementation was found to significantly increase wool production and sulfur concentration in wool (Sherlock et al., 2001). Moreover, wool fiber cystine content was directly proportional to the dietary SAA levels and thus to the wool quality. Moreover, daily supplementation with 2 g of cysteine or 2.46 g of methionine improved wool production per unit area of skin by 35–130% and wool sulfur content by 24–35% (Reis and Schinekel, 1961; Reis and Schinckel, 1963). Nutritional supply of SAAs significantly alters the mitotic rate of hair follicle bulb cells: higher SAA supplementation leads to increased proliferation and differentiation of follicle bulb cells and further keratinization (Hynd, 1989). Cellular uptake of cysteine is mediated by various transporters, often with tissue-specific distribution. Downes et al. (1962) studied cysteine uptake in human hair follicles and outer root sheath cells *ex vivo* and found that fibroblasts demonstrated cysteine uptake and that the cysteine transporter ASC was present in hair follicles and outer root sheath cells. Cysteine is also the main component of keratinized proteins in feathers, and in birds, the demand for cysteine is fulfilled via the transsulfuration pathway. Inhibition of the transsulfuration pathway can affect hair follicle development, skin thickness, and growth in broilers; moreover, this inhibition is associated with the upregulation of hepatic cystathionine synthase and cystathionine lyase mRNAs (Silva et al., 2018). Silva et al. (2019) randomized chicks into control and cysteine-deficient groups for 49 days and measured their skin layer thickness and hair follicle length and thickness on days 10, 24, 34, and 49 after treatment, respectively. Chicken epidermis in the cysteine-deficient group had thinner and shorter hair follicles with increased cystathionine synthase and cystathionine lyase mRNA expression—indicating a disruption in the transsulfuration pathway (Silva et al., 2019).

Sulfur metabolism is interwoven through various life processes, where it contributes to their vitality. Ongoing studies on the role of sulfur in goat wool fiber growth are mainly focused on sulfur’s nutritional effect, its suitable dietary level, and the nitrogen–sulfur ratio, but without much attention to the underlying molecular mechanisms. Wang et al. (2006) found that related hormones changed in cashmere goats after melatonin implantation and that it regulated nitrogen distribution in the body and the villus, thus promoting cashmere wool growth. Sulfur metabolism is closely related to nitrogen metabolism (Wang and Jia, 1999).

To date, the knowledge on the expression pattern of sulfur metabolism genes in cashmere goats remains limited. In this study, we hypothesize that sulfur metabolism genes interact with melatonin to regulate villus growth. Here, high-sulfur protein family genes, sulfur metabolism genes, and melatonin in cashmere goats are used as entry points to study the important genes involved in the regulation of villus growth, eventually elucidating the underlying signal transduction networks.

## Materials and Methods

### Experimental Animals and Sample Collection

Six 16-month-old female cashmere goats from Hanshan, Inner Mongolia were included in this study. They were divided into two groups (*n* = 3 in each group): melatonin implantation (treatment group) and melatonin non-implantation (control group). In total, we collected 72 blood and 72 skin samples over 12 months (12 each from each goat).

During the experiment, 2 mg/kg body weight melatonin was implanted subcutaneously behind the ears of the treatment group goats, whereas no melatonin was implanted in the control group. One month after implantation, scapula skin samples (size, 1 cm^2^) and venous blood samples were collected and quickly frozen and stored at -80°C in liquid nitrogen. The melatonin concentration in plasma were tested (Supplementary Figure 1).

Moreover, primary follicles (PFs) and secondary follicles (SFs) were collected in each month from four female cashmere goats from Inner Mongolia. PF samples were collected from 12 months of the year and SF samples were collected from August to February of the following year. These PFs and SFs were plucked from the side of the torso of the goats at the beginning of each month. First, the cashmere and wool were separated by careful observation, and then the cashmere and wool were quickly pulled out from the torso of the goats. Use chloroform sterilized scissors to cut the hair follicles 1 cm away from the root of the cashmere and wool. The PF and SFs were immediately frozen without any chemical solutions in liquid nitrogen for storage and transported until RNA isolation. In total, 12 PFs and 8 SFs samples were collected.

### RNA-seq Library Construction and Sequencing Using Blood and Skin Samples

Total RNA was extracted from all blood and skin samples using TRIzol reagent (TaKaRa), according to the manufacturer’s instructions. The blood and skin samples at –80°C were thawed (skin material was ground in liquid nitrogen). 1 ml TRIzol reagent was added and thoroughly mixed. The supernatants were obtained after the samples were centrifuged at 12,000 *g*, for 10 min at 4°C. The top layer was collected after chloroform was added. A 1:1 V of isopropanol was added to each tube before being centrifuged at 12,000 *g* for 10 min at 4°C. The supernatants were discarded and the remaining pellets were washed with 1 ml of 75% ethanol and centrifuge at 7500 *g* for 5 min. All of the residual ethanol was then removed and the pellets were allowed to air dry for 4 min. The RNA samples were then redissolved in 30 μl of DEPC (Diethyl Pyrocarboanate) treated H2O and stored at –80°C. After the RNA samples were quantitated, mRNAs was enriched with oligo (DT) magnetic beads. Then, the fragment buffer was added to break the mRNA into shorter fragments. We used the mRNA as the template, single-stranded cDNA was synthesized using a randomized hexamer, followed by the synthesis of double-stranded cDNA by adding buffer, dNTPs, DNA polymerase I, and RNase H. The double-stranded cDNA was then purified using AMPure XP beads, and then the second strand of cDNA containing U was degraded using USER enzyme. Purified double-stranded cDNA was then repaired at the end, followed by the addition of a tail and connection to the sequencing connector. Next, AMPure XP beads were used to select the fragment size. Finally, the final library was obtained via PCR amplification and purification. After RNA-seq library construction, Qubit 2.0 was used for preliminary quantification, followed by library insert size detection using Agilent 2100. After the inserts met the expectations, Q-PCR was used to accurately quantify the effective concentration of the library and ensure the quality of the library. In total, 72 libraries from skin samples and 72 libraries from blood samples have been constructed.

### Single-Molecule Long-Read Sequencing and Optimization of Hair Follicle Gene Structure in Cashmere Goat

Total RNA used for constructing an Iso-Seq library: mRNA was reverse-transcribed and then amplified using SMARTer PCR cDNA Synthesis Kit and PrimeSTAR GXL DNA Polymerase. The subsequent library construction was performed using the SMRTbell Template Prep Kit 1.0, and its library sequencing was performed on a PacBio RS II. The full-length transcriptome was analyzed using SMRTLink; it mainly included three stages: CCS acquisition, classification, and clustering. We used the Quiver algorithm in combination with the non-full-length sequences to polishing the consensus isoforms obtained through clustering and screen high-quality (HQ) and low-quality (LQ) sequences for subsequent analysis. Based on the full-length transcripts after polishing (HQ + LQ), the sequences were aligned to the reference genome (Capra hircus ARS1) by using the alignment software program Gmap (Wu and Watanabe, 2005). Only the sequences with identity > 0.9 and coverage > 0.85 in the alignment results were selected. The sequences with differences in the last exon at the 5′ end were merged using ToFU for collapse (with the options: –dun-merge-5-shorter). The GFF annotation file of the collapsed transcript was compared with that of the reference genome using gffcompare. The start and end positions of known genes were also optimized.

### DEG Analysis

High-quality clean data obtained by filtering data according to strict standards can be used for further research and publication. When the N content in any sequencing read is >10% of the base number of the read, the paired reads should be removed. Moreover, when the LQ base number (Q ≤ 5) base present in any sequencing read is >50% of the base number of the read, the paired reads should be removed. We used HISAT to match the read segments to the reference genome, which was obtained from the goat reference genome and its corresponding genome annotation file in the NCBI database (*ARS1*) (Dong et al., 2013). DEGs were detected using limma in the R package (Ritchie et al., 2015) with default parameters to screen out DEGs according to FPKM. The genes with |log_2_fc| > 1 and *P* < 0.05 were identified as DEGs.

### Weight Gene Coexpression Network and ASE Analyses

In the control group, 86 sulfur metabolism genes were differentially expressed in the blood and skin, whereas 4839 genes were coexpressed. A coexpression network was thus constructed using the weight gene coexpression network analysis (WGCNA) (Langfelder and Horvath, 2008) in R package for 4839 genes with 86 sulfur metabolism functional DEGs used as traits. The parameter β was given a default value from 1 to 30, as determined by the function SFT $powerestimate, with the minimum number of modules chosen to be 30.

BAM files were single-nucleotide polymorphisms (SNPs) called with samtools to generate BCF files, which were converted to VCF files using bcftools, containing genotype data for individuals. Heterozygous SNPs in an individual were filtered with the criteria that the individual’s genotype was heterozygous, the coverage was >30 in both REF and ALT reads for both individual and SNP sites in a month, and finally, high confidence expressed SNPs were obtained.

Next, for ASE, we defined a haplotype concordant with the reference genome as ref allele and its homologous chromosome as ALT allele. The probability of occurrence of each allele was calculated via binomial distribution. A corresponding *P*-value of ≤0.05 was considered to indicate significance.

## Results

### Genome-Wide Identification, Characterization, and Expression Profile of High-Sulfur Genes in Cashmere Goats

**FIGURE 1:**
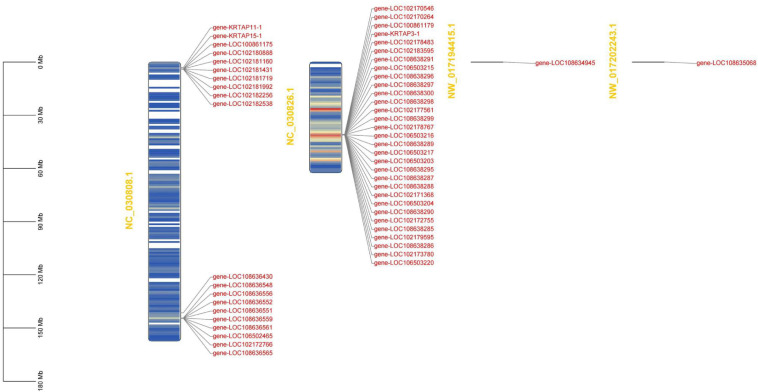
Chromosomal mapping of 53 high-sulfur protein genes.

To run a complete search for identifying high-sulfur genes in the goat genome, two methods were used: We first searched the high-sulfur for protein sequences based on the published high-sulfur protein sequences in human (Khan et al., 2014) and sheep (Gong et al., 2016) and the Hidden Markov Model. For the sequence not found using the first method, we used the second method, where the published human and sheep high-sulfur protein sequences were employed as a query to perform blast against the goat reference proteome with identity score ranked first, alignment length equal to sequence length, and mismatches of <5. In total, 53 genes were assigned as high-sulfur protein genes in cashmere goats (Supplementary Table 1). All the high-sulfur protein genes are distributed in the 3M–4M and 144M regions of goat chromosome 1 and the 40M–41M region of goat chromosome 19 (Figure 1).

**FIGURE 2:**
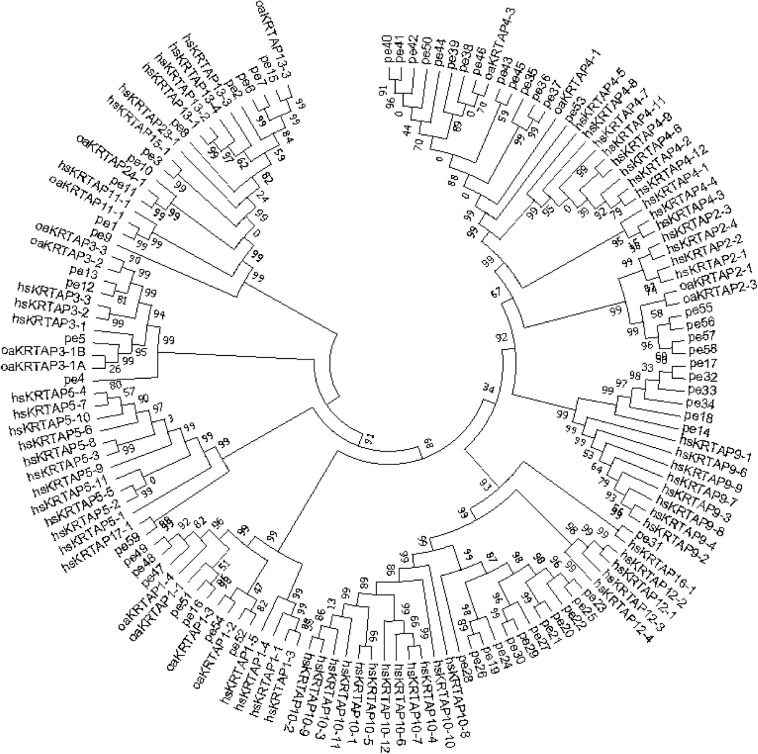
Phylogenetic tree of human, sheep, and goat high-sulfur proteins. The prefixes pe, oa, and hs indicate goat, sheep, and human, respectively.

Single-molecule long-read sequencing of hair type in cashmere goat transcriptome data was used to complement and correct the 35 high-sulfur protein gene sequences in the cashmere goat. Consequently, 35 high-sulfur protein genes with relatively complete and accurate sequences were finally obtained (Supplementary Table 2). To classify the high-sulfur protein gene family in the cashmere goat, we built a phylogenetic tree using all the 53 high-sulfur protein sequences in cashmere goats, humans, and sheep using the NJ method. This tree illustrated that high-sulfur proteins in the cashmere goat can be divided into 12 subfamilies (Figure 2). We designated these subfamilies according to the human and sheep high-sulfur protein classification. No members were present in the *KRTAP*5, *KRTAP*23, and *KRTAP*12 subfamily. In contrast, 7, 4, 4, 14, 6, 12, 2, 5, 1, 1, 1, and 2, high-sulfur proteins were found to belong to the *KRTAP*1, *KRTAP*2, *KRTAP*3, *KRTAP*4, *KRTAP*9, *KRTAP*10, *KRTAP*11, *KRTAP*13, *KRTAP*15, *KRTAP*16, *KRTAP*17, and *KRTAP*24 subfamilies, respectively.

**FIGURE 3:**
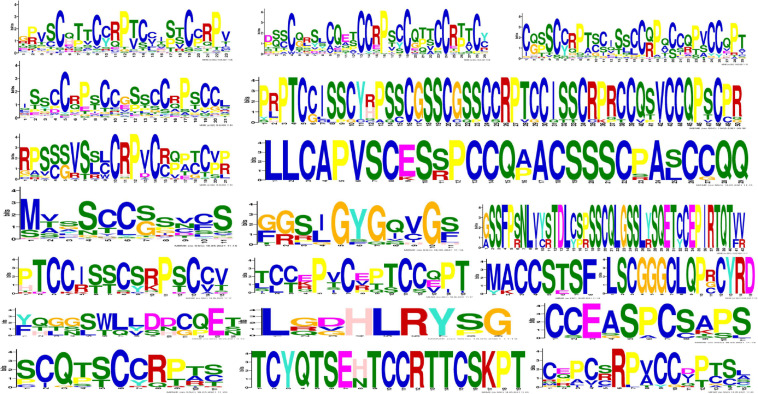
Amino acid frequency of 20 conserved motifs. Each letter represents an amino acid; the larger the letter in a motif, the higher is amino acid frequency.

**FIGURE 4:**
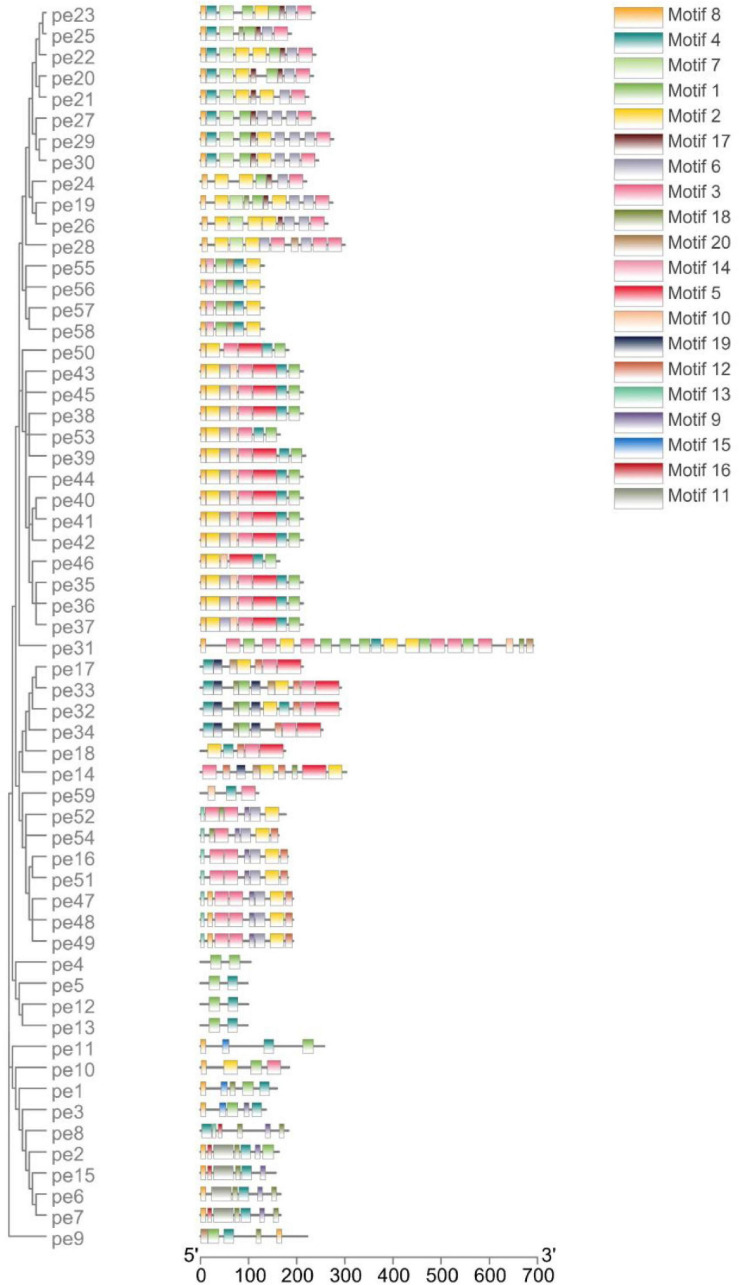
Distribution of conserved motifs of high-sulfur protein genes.

The conserved motifs in the high-sulfur proteins were further investigated using the online tool MEME (Bailey et al., 2009), and a total of 20 conserved motifs were detected (Supplementary Table 3). The height of each stacked letter in Figure 3 represents the probability that the amino acid appears at the site, with a high frequency of cysteine (Figure 3). The structures of the motifs demonstrated that the motifs are conserved within each subfamily—indicating that the gene function within the families was quite conserved. Of them, the motifs 8, 4, and 7 were conserved in *KRTAP*10. Moreover, motifs 4, 13, 1, and 8 were conserved in *KATAP*9, *KATAP*1, *KRTAP*3, and *KRTAP*13, respectively (Figure 4).

**FIGURE 5:**
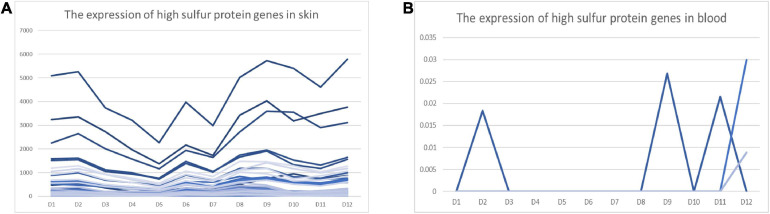
FPKM of high-sulfur protein genes over the 12 study months. Abscissa represents month, and ordinate represents FPKM. D represents the control group.

We found that 53 high-sulfur protein genes were expressed in skin variably over 1 year. In contrast, only three genes—namely *KRTAP11-1*, *LOC102170546*, and *LOC108638285*—were expressed in blood, but at a low level (Figure 5B). The expression of high-sulfur protein genes in the skin demonstrated a decreasing trend from February to May, followed by an increasing trend from June to September (Figure 5A).

**FIGURE 6:**
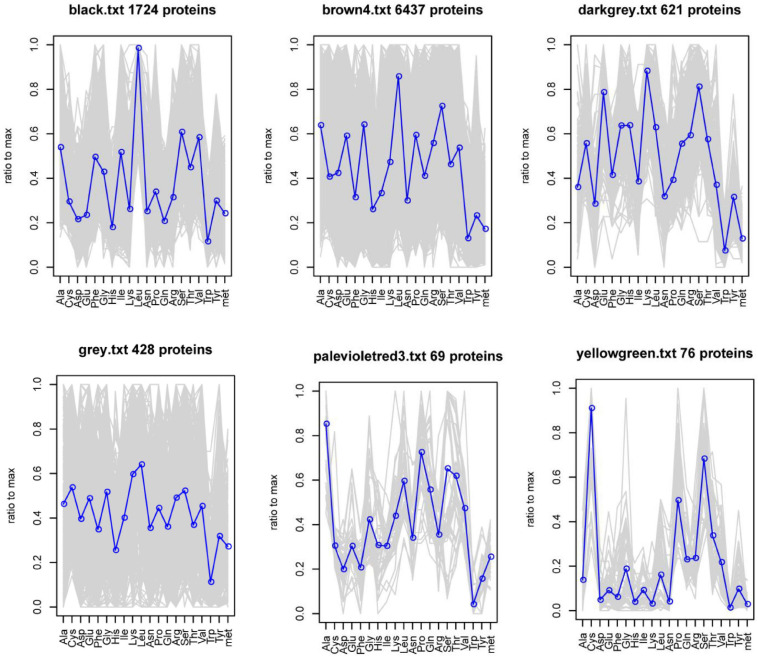
Abundance of 20 amino acids in each CDS in the module.

Weight gene coexpression network analysis was used to identify the coding sequences (CDS) with coordinated amino acid expression patterns. Here, we divided the 9355 CDS (Cys ≥ 3) of the goat reference proteome into six modules. The number of CDS in each module was 1724, 6437, 621, 428, 69, and 76, and a total of 5160 genes were annotated. Of these, 52 high-sulfur protein genes were annotated in the yellow-green module, and the protein amino acid expression pattern was found to be different from that of other genes. Moreover, cysteine abundance was noted to be highest, followed by that of serine (Figure 6).

### Profiling High-Sulfur Protein Gene ASE in Cashmere Goats

**FIGURE 7:**
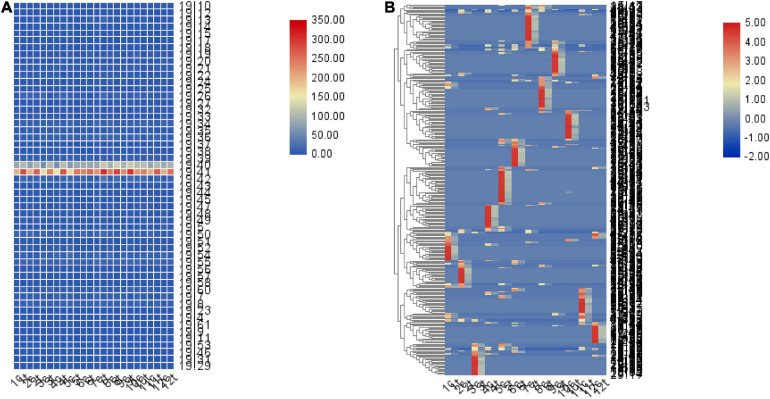
Heat map of the number of ASE loci in the 1M region. **(A)** Quantitative heat map of ASE loci in every 1M region of chromosome 19. **(B)** A total of 308 regions exhibiting differences in the proportion of ASE sites between the control and treatment groups (minimum/maximum ≤ 0.3), and the locations at which ASE numbers differed between months. The number represents the month, c represents the control group, t represents the treatment group.

Gene expression profiling using RNA-seq and genotyping were performed on data. We identified 172771 SNPs. We further counted the number of ASE per 1M region of chromosome 19. Moreover, TBtools (Chen et al., 2020) was used to display it in the form of a heat map. The results indicated a higher number of ASE in the 40–41M region of chromosome 19 compared with other regions (Figure 7A). The number of high-sulfur protein genes with ASE ranged from 35 to 43 for all months of the year in the control group (Supplementary Table 4) and from 37 to 45 for all months of the year in the treatment group (Supplementary Table 5). In total, 308 regions with different numbers of ASE between the control and treatment groups were detected; here, the region of difference number ASE was defined as the ratio of the two groups (min/max) ≤ 0.3. The locations at which ASE occurred differed between the 12 months (Figure 7B). The total number of ASE sites was higher in the control group than in the treatment group, indicating that melatonin inhibited ASE. Moreover, annotation revealed that these regions contained 856 genes, of which 16 genes play important roles in the Hippo signaling pathway (*P* = 0.008; Supplementary Figure 2). Furthermore, 37 transcription factors and 60 transcription cofactors were scattered throughout these regions.

After melatonin implantation, the expression of 26 transcription cofactors and 22 transcription factors changed, all of which showed higher expression levels in the control group from August to December. Moreover, the expression pattern changed in the treatment group, with elevated expression levels in May and April (Supplementary Figure 3).

**FIGURE 8:**
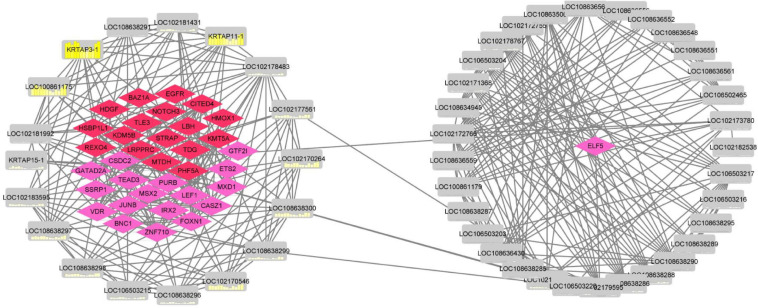
Coexpression regulatory network of high-sulfur protein gene, transcription factor, and transcription cofactor. The red node represents transcription factor, the pink node represents transcription factor cofactor, and the gray node represents high sulfur protein gene. The yellow node shows the expression over 12 months.

In total, 47 high-sulfur protein genes were used to construct a coexpression network using Pearson’s correlation with a cutoff (|adj|≥ 0.98, *P* ≤ 0.01; Figure 8). We found that the regulation between 18 high-sulfur protein genes and 17 transcription factors as well as 18 transcription factor cofactor genes was relatively more complex. Moreover, 29 high-sulfur protein genes and *ELF*5 (the key gene of the regulatory network) were noted to constitute a regulatory network. Pearson’s correlation analysis revealed positive regulatory relationships between the high-sulfur proteins and all the transcription factors and cofactors.

### Identification of Sulfur Metabolism Gene Expression Patterns in Cashmere Goat Blood and Skin

We, next, ran a complete search for sulfur metabolism genes in the cashmere goat genome using all the annotated pathway of the sulfur metabolism from GO database^1^. In total, 29 species were found to cover 116 sulfur metabolism pathways with a total of 1789 sulfur metabolism genes, including 321 sulfur metabolism genes in the cashmere goat. In total, 10, 1, and 310 sulfur metabolism genes were specifically expressed in the skin, in the blood, and in both the skin and blood, respectively.

**FIGURE 9:**
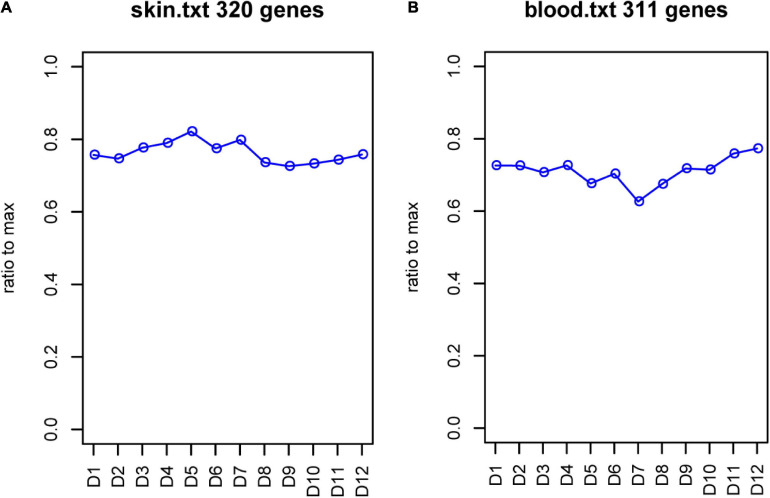
FPKM of sulfur metabolism genes. **(A)** FPKM of sulfur metabolism genes in skin. **(B)** FPKM of sulfur metabolism genes in blood. Abscissa represents month, and ordinate represents ratio to max. D represents the control group.

In total, 20 GO terms incorporating the cofactor metabolic process, sulfur compound biosynthetic and metabolic process, acyl-CoA metabolic process, mucopolysaccharide metabolic process, and sulfur compound catabolic process were enriched significantly (Supplementary Figure 4). For sulfur metabolism genes, the expression patterns may play important roles in the growth of different wools. Therefore, we analyzed sulfur metabolism gene expression all year round. The results showed that changes in the blood and skin mainly occurred between June and July. In the skin, a total of 320 genes expression showed a significantly elevated trend from January to June, with a slow decrease in expression after July. In the blood, the expression of 311 sulfur metabolism genes showed an overall decreasing trend between January and June, with the lowest expression in June and a gradual increase after July (Figure 9).

We then investigated the transcriptional differences that characterize preferentially or specifically expressed genes in each development stage of skin and blood. Over the year, the skin had 43, 40, 37, 46, 43, 46, 45, 41, 39, 43, 36, and 39 genes with upregulated expression and had 16, 12, 8, 8, 9, 12, 11, 14, 13, 12, 16, and 14 genes with downregulated expression compared with that in blood (Supplementary Table 6). Over the study months, the number of tissue-specific genes varied between 3 and 9, with a total of 15 genes showing tissue-specific expression. These results suggested the potential function of sulfur metabolism genes have important roles in cashmere goat skin.

### Global Transcriptome Analysis for Tissue-Specific Functional Pathway

The transcriptome analysis of the blood and skin tissues at different development stages of the control and treatment groups was performed to provide crucial system-level insights into molecular mechanisms underlying wool and cashmere development and melatonin. The transcriptome data from skin and blood samples were 99.09% (Supplementary Table 7) and 98.55% (Supplementary Table 8) valid, respectively. In total, 23,108 and 23,050 genes were identified in the skin of the control and treatment groups, respectively. Moreover, 21,378 and 21,304 genes were identified in the blood of the control and treatment groups, respectively. Furthermore, 56–57% and 59–60% of genes exhibited medium expression level (FPKM = 5–100) in skin and blood of the different groups, respectively.

We then analyzed the changes in gene expression associated with treatment and control for different tissues (blood vs. skin). There were 6,327 DEGs in the control group (|log_2_fc| > 1, *P* < 0.05), with 3,715 genes upregulated in the skin and 2,612 genes upregulated in the blood. The number of upregulated DEGs in the treatment group was 3634 in the skin and 2607 in the blood.

**FIGURE 10:**
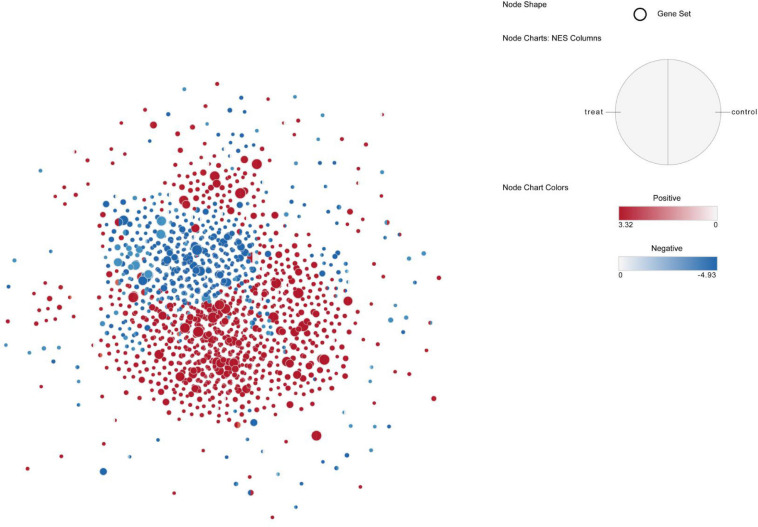
Up-regulated pathway network of blood and skin in control and treat, the red node indicates that the pathway is up-regulated in the skin, and the blue node indicates that the pathway is up-regulated in the blood, the left half of the node represents treat and the right side represents control.

Gene set enrichment analysis (GSEA) was then used to find enriched GO gene-sets upregulated in the blood and skin of the treatment and control groups. The results were generated after scoring DEGs using the Signal2Noise statistic. Only gene-sets that passed conservative significance thresholds (FDR < 25%) were selected for display in the Enrichment Map; consequently, 780 and 504 gene-sets were enriched in the skin and blood of the control group, respectively, and 824 and 504 gene-sets were enriched in the skin and blood of the treatment group, respectively. Of all the pathways enriched by the DEGs in control (blood vs. skin) and treatment (blood vs. skin) groups, 522 functional pathways of cell development regulation were strongly correlated with each other; moreover, these pathways were correlated with the WNT canonical pathway, BMP signaling pathway, cell motility, kinase activity, and muscle tissue development activity (Supplementary Figure 5A). This two-enrichment visualization demonstrated the same (all red or all blue) or different enrichment across the two data sets. Here, we noted that the agreement between the control and treatment groups was noted at very high-most nodes, which are all one color, thus indicating that these gene-sets have tissue-specific upregulation (Figure 10). In the treatment group, 11 gene-sets were significantly enriched in the blood but not in the skin, and gene-sets with stronger upregulation only in the skin were present in nine functional pathways (Supplementary Figure 5B). In total, 10 gene-sets were enriched in the control group and only constituted a minor portion of the blood map. Moreover, gene-sets with stronger enrichment only in the skin were present in 12 functional pathways (Supplementary Figure 5C). In total, 15 functional pathways in the control group were upregulated in the blood, but an opposite result was noted in the treatment group, and up regulated in the skin (Supplementary Figure 5D). Actin filament organization, GTPase binding, rho GTPase binding, ruffle membrane, and small GTPase-mediated signal transduction gene-set were strongly induced in the blood after melatonin treatment. Thus, these five functional pathways could be more dependent on melatonin in the blood (Supplementary Figure 5D).

### Effect of Melatonin on Sulfur Metabolism Gene and Tissue-Specific Gene Expression Changes

**FIGURE 11:**
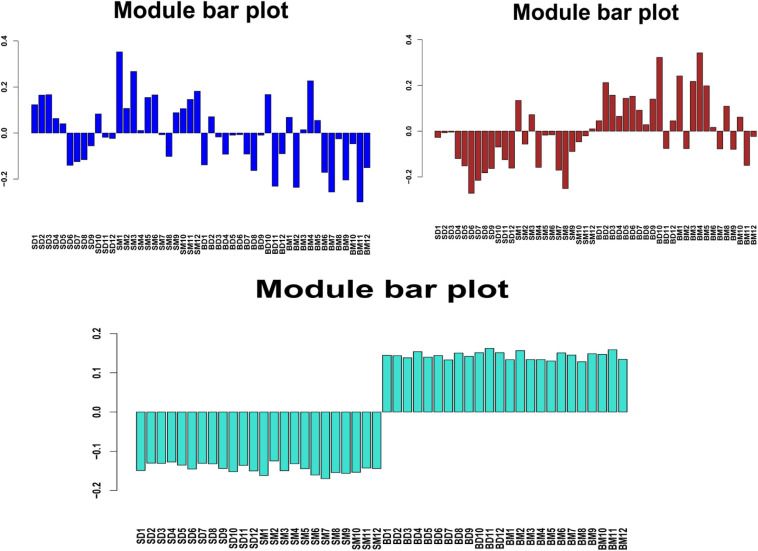
Block eigenvalue bar chart. SD and SM represents the skin samples in the control and treatment groups, respectively, and BD and BM represents the blood samples in the control and treatment groups, respectively.

To understand the tissue-specific expression patterns of all genes, we clustered all their expression patterns (4389 genes) by using WGCNA. We identified three main gene modules, where the turquoise modules represent the tissue-specific expression patterns (Figure 11), including 3629 genes. This module demonstrated a more pronounced and patterned regulation by tissues but were less patterned by development stages.

**FIGURE 12:**
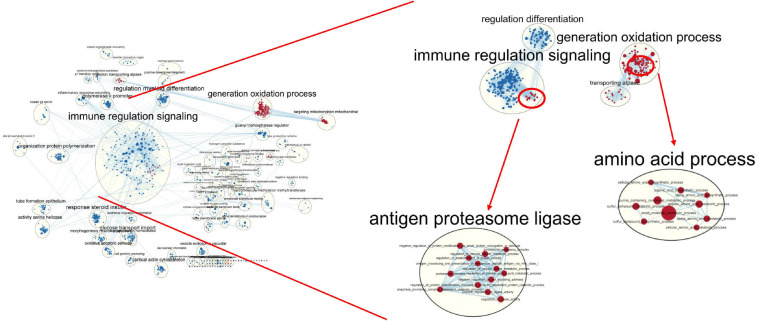
Global network of enrichment results of turquoise module genes. The red and blue nodes indicate that the pathway is upregulated in the skin and blood, respectively.

Gene set enrichment analysis (Subramanian et al., 2005) and Enrichment Map (Daniele et al., 2010) were used to analyze upregulated pathway including the turquoise module genes in the blood and skin. The results showed that many of the key pathways in the turquoise module were also tissue-specific (Figure 12). In the skin, 168 upregulated pathways were enriched, including sulfur compound metabolic, cellular amino acid catabolic, organic acid biosynthetic, and WNT signaling pathways. There were 687 upregulated pathways in the blood, including adaptive immune response, innate immune response, immune system development, and regulation of leukocyte migration. The network simultaneously showed the pathways upregulated in the blood and skin; here, blue and red nodes denoted the upregulated pathways in the blood and skin, respectively. Most nodes of the node were of one color, whereas the remaining pathways were both blue and red, demonstrating that a subset of gene-sets both regulated metabolic processes in the blood and skin.

The enriched pathways in blood appeared in most functional groups (blue nodes), indicating that the response of blood to the expression of genes with tissue-specific expression patterns was relatively strong. The immune-related pathways were significantly upregulated in the blood, whereas the oxidation-related pathways were significantly upregulated in the skin.

In general, there were fewer pathways upregulated in the skin than in the blood (168 vs. 687). Many of the gene-sets upregulated only in the skin were associated with amino acid metabolism, sulfur compound biosynthesis, and transport kinase function. The tissue-specific modules were positively or negatively correlated with 86 sulfur metabolism genes, with a correlation coefficient of >9.8; moreover, the correlation coefficient of individual module was 0.99 (*P* < 1e^–20^), indicating that the module obtained via WGCNA was high reliable (Supplementary Figure 6).

According to the WGCNA results, we constructed coexpression modules, and part of the genes in the tissue-specific module involved in pathway specificity were upregulated in the skin, such as those related to amino acid metabolism, sulfur compound biosynthesis, and transport kinase function. Because various biological metabolic processes are not caused by a single factor, the development of gene interaction networks not only provides an effective way to probe complex metabolic processes but also improves the comprehensiveness of our research. Based on this, we constructed an interactive regulatory network between the tissue-specific genes and sulfur metabolism genes.

**FIGURE 13:**
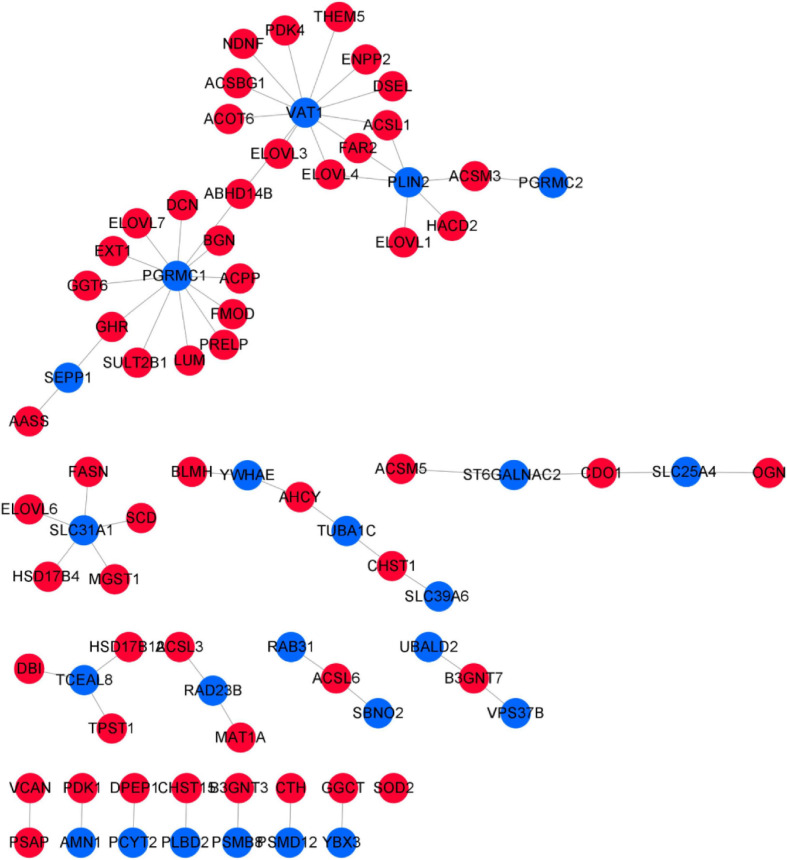
Correlation network of sulfur metabolism genes and tissue-specific expressed genes. The blue nodes represent tissue-specific genes, and the red nodes represent sulfur metabolism genes.

For network construction, genes were screened from the turquoise module and linear discriminant analysis effect size (LEfSe) (Segata et al., 2011) was used to elucidate tissue-specific genes for network construction. LEfSe, which judged the factors most likely to explain the different categories, was used to search for biomarkers. Filtering was performed on the basis of the GS of genes and phenotypic traits in the WGCNA results, and genes with a GS of top3 were selected as positively correlated. In total, 258 relation pairs were obtained with GS ≥ 0.85 and *P* ≤ 1e^15^ involving 122 turquoise genes. LEfSe results illustrated 23 genes with tissue-specific expression in the treatment group, but no genes with tissue-specific expression were noted in the control group (Supplementary Figure 7). Based on the 23 genes with tissue-specific expression and the sulfur metabolism gene regulatory network, *VAT*1, *PGRMC*1, and *SLC31A*1 were found to be hub genes (Figure 13)—all of which were highly and less expressed in the skin and blood of the treatment group, respectively.

Although we constructed coexpression modules between the tissue-specific genes and sulfur metabolism genes, the specific biological function of these genes was unknown. By using Database for Annotation, Visualization and Integrated Discovery (DAVID, version 6.7) (Huang et al., 2009), we performed functional enrichment analysis for all genes in the regulatory network. The results indicated that genes in the regulatory network were enriched to a result closely related to the tissue biological functions. For instance, the tissue-specific gene *VAT*1—highly expressed specifically in skin tissue after the implantation of melatonin—is mainly involved in the oxidation–reduction process, and regulates 11 genes involved in fatty acid metabolism (*THEM*5, *ENPP*2, *DSEL*, *ACSL*1, *FAR*2, *ELOVL*4, *ELOVL*3, *ACOT*6, *ACSBG*1, *NDNF*, and *PDK*4). There are four genes involved in cysteine and SAA metabolism (*CTH*, *CDO*1, *AHCY*, and *MAT1A*). Among these, *CTH* interacts with *PSMD*12, *CDO*1 with *ST6GALNAC2* and *SLC25A4*, *AHCY* with *YWHAE* and *TUBA1C*, and *MAT1A* with *RAD23B*. After melatonin implantation, *PSMD*12, *ST6GALNAC2*, *SLC25A4*, *TUBA1C*, and *RAD23B* were upregulated in the skin. The function of these genes was most related to cell energy metabolism and cell cycle.

## Discussion

Sulfur is an essential mineral for organisms it not only functions as a structural component but also performs specific functions in cellular metabolism (Mcnab et al., 1990). In sheep, the high-sulfur protein genes family comprises six members; of these, *KRTAP*4 and *KRTAP*5 are the ultra–high-sulfur protein genes, containing eight members in total: *KRTAP*4-1, *KRTAP*4-2, *KRTAP*4-3, *KRTAP*5-1, *KRTAP*5-2, *KRTAP*5-3, *KRTAP*5-4, and *KRTAP*5-5 (Gong et al., 2016). The high and ultra–high-sulfur protein genes are located on human chromosomes 17q21.2, 21q22.1, 21q22.3, 11p15.5, and 11q13.4 (Rogers et al., 2006).

Studies have shown that in sheep, sulfur and SAAs play a specific role in metabolism; the sulfur levels in erythrocytes and plasma proteins (mainly those containing cystine and methionine) are higher in sheep than in cattle, horses, and dogs (MaπκoBa and Wang, 1954). Cystine metabolized from glutathione in blood is a constituent of wool proteins (MaπκoBa and Wang, 1954). Then, the sulfur metabolism during the enrichment of sulfur from the blood to the skin is extremely important. Here, we performed deep transcriptome sequencing on samples obtained from cashmere goat blood and skin; moreover, the expression patterns of high-sulfur protein genes were systematically analyzed to provide further insights into the molecular mechanism underlying sulfur’s role in cashmere goats’ wool growth regulation.

Significant (*P* ≤ 0.01) interactions were noted between 47 high-sulfur protein genes and transcription factors and cofactors with ASE. However, the ASE of these transcription factors and cofactors was inhibited by melatonin. Thirteen genes in the interaction network were functionally associated with the functional of hair growth.

*Elf*5 (Driskell et al., 2013) and *EGR*1 (Phan et al., 2020) directly regulate *KRT*18 expression, and *Elf*5 may play a role in mediating fibroblast growth factor regulatory processes. *LEF*1 in fibroblasts can promote skin repair and hair follicle regeneration, and it is a key effector in the WNT signaling pathway involved in hair follicle morphogenesis. WNT target gene transcription is repressed by *TLE*3, and β-catenin directly competes with TLE proteins for TCF/LEF binding to complete gene regulation at WNT pathway activation.

β-Catenin inhibition by *VDR* promotes keratinocyte proliferation and hair follicle differentiation (Hu et al., 2014). *JUB* via WNT/β-catenin signaling can induce epithelial–mesenchymal transition in liver cancer cells (Wang et al., 2018). Loss of functional FOXN1 also has profound effects on epidermal keratinization and epidermal adhesion (Mecklenburg et al., 2010). Mutant *GTF2I* can induce thymic epithelial cell transformation and metabolic alterations (Kim et al., 2020). TGF-β regulates many cellular processes, including cell proliferation and differentiation. Moreover, STRAP is a TGF-β inhibitor of signaling and an important regulator of cell proliferation. TGF-β receptors I and II interact and negatively regulate TGF-β-induced gene expression (Kim et al., 2007). Furthermore, MSX2 is involved in keratin formation (Ma et al., 2003). Downregulation of KHSRP expression in the skin can inhibit mir-198 expression and enhance FSTL1 expression, which stimulates keratinocyte migration (Briata et al., 2016). *KDM5B* is important in cell differentiation, stem cell self-renewal, and other developmental processes (Han et al., 2016). *TEAD*3 can promote human epidermal growth (Li et al., 2020).

In general, mice lacking EGFR fail to develop hair (Tripurani et al., 2018). Thus, it is speculated that the interaction of transcription factors and cofactors with high-sulfur protein genes is upregulated to promote villus growth after melatonin implantation, further enriching current knowledge on high-sulfur protein gene family functions.

In addition to providing sulfur required for maintaining redox reaction, the metabolism of these SAAs produces proteins with a role in maintaining the spatial structure of hair, where intermediary metabolites mediate intercellular and intracellular signaling, thus promoting epigenetic regulation of gene expression and collectively contributing to hair growth (Ward and Denicola, 2019).

With regard to the underlying mechanism of sulfur, we first identified 321 sulfur metabolism genes in the cashmere goat and observed the expression of these genes in skin and blood tissues. Sulfur metabolism gene expression changes in the blood and skin mainly occurred between June and July, and the number of sulfur metabolism genes in the skin was significantly higher than that in the blood. These results suggested a potential function of sulfur metabolism genes which may have important roles to the skin of the cashmere goats. The skin has been highlighted as valuable candidate tissues for research transport system for cysteine (Thomas et al., 2007). Evidence from the coexpression network analysis revealed the sulfur metabolism genes associated with 23 tissue-specific genes, which were only upregulated in the skin or blood after melatonin implantation. For instance, *CTH*, *CDO*1, *AHCY*, and *MAT1A* were found to be involved in cysteine and SAA metabolism. *CTH* interacts with *PSMD*12, *CDO*1 with *ST6GALNAC2* and *SLC25A4*, *AHCY* with *YWHAE* and *TUBA1C*, and *MAT1A* with *RAD23B.*

Moreover, *PSMD*12 plays a key role in regulating the cell cycle, DNA damage repair, and apoptosis (Saez and Vilchez, 2014; Küry et al., 2017; Contreras et al., 2018; Okumura et al., 2018). *PSMD*12 knockdown can inhibit cell growth and migration (Du et al., 2020). *ST6GALNAC2* has functions in the PI3K/AKT pathway (Ren et al., 2014), which is involved in many cellular processes, including proliferation, differentiation, apoptosis, cell cycle progression, and cell motility (Bellacosa et al., 2005; Engelman et al., 2006). *SLC25A4* has been implicated to have many transport functions for various molecules on the mitochondrial membrane, including ATP/ADP and amino acids, which are molecules constituting cellular energy sources (Albahde et al., 2020).

*TUBA1C* overexpression is mainly associated with cell cycle regulation. Yin et al. (2019) found that *YWHAE* and *TUBA1C* are functionally related in keratinocytes. We also found that *YWHAE* interacts with *TUBA1C* and both genes were specifically upregulated in the skin after melatonin treatment. Notably, *PSMD*12, *ST6GALNAC2*, *SLC25A4*, *YWHAE*, *TUBA1C*, and *RAD23B* were all upregulated specifically in the skin after melatonin implantation, and the functions of these genes were mostly related to cellular energy metabolism and cell cycle. The energy required for cashmere wool growth comes from a series of oxidation–reduction reactions during cellular metabolism, which are facilitated by different molecules with constituent sulfur atoms (Thomas et al., 2007). The tissue-specific gene *VAT*1 was highly and specifically expressed in the skin tissue after melatonin implantation. This gene is mainly involved in redox processes and regulates 11 genes involved in fatty acid metabolism: *THEM*5, *ENPP*2, *DSEL*, *ACSL1*, *FAR*2, *ELOVL*4, *ELOVL*3, *ACOT*6, *ACSBG1*, *NDNF*, and *PDK*4. These results highlight the potential role of melatonin in skin in upregulated expression of genes involved in cellular energy metabolism as well as those involved in cell cycle and activation of sulfur metabolic processes provides sufficient SAAs for cashmere wool development.

Intracellular targets where melatonin exerts biological actions are receptors, and they are divided into membrane receptors [melatonin type 1 (MT1) and type 2 (MT2) receptors] and nuclear receptors (RORα, RORβ, and RORγ) (Liu et al., 2016). MT1 and MT2 receptors have been detected in mammalian skin (Söderquist et al., 2015). Human skin primarily transcribes *MT1*, whereas *MT*2 is expressed along with *MT1* in specific conditions. In contrast, mouse skin shows exclusive expression of *MT*2. In human skin, *MT*1 has been detected in the differentiated layer of the epidermis, the outer and inner root sheaths of HF, sweat glands, and blood vessels, whereas *MT2* has been detected in the inner root sheath, sweat glands, and blood vessels (Slominski et al., 2018). In contrast, cashmere goat skin tissues showed no expression of membrane receptors. Expression of only *ROR*α, a nuclear receptor, was detected in the skin of cashmere goat (Wang, 2016).

Based on the overall panoramic regulatory network, we concluded that melatonin affects the pathways involved in the DEG between blood and skin, and the pathways demonstrated spatially specific upregulation trends; for example, after melatonin implantation, the ErbB signaling pathway was found to be upregulated only in the blood from control cashmere goats, whereas only the Notch signaling pathway was noted to be upregulated only in the treated cashmere goat skin.

## Conclusion

In total, 53 high-sulfur protein genes and 321 sulfur metabolism genes were identified and further analyzed for their expression in the skin and blood tissue. These high-sulfur protein genes are distributed in the 3–4M and 144M regions of chromosome 1 and the 40–41M region of chromosome 19 in goats. We found that ASE in the 40–41M region of chromosome 19 was significantly higher than that in the other regions over the entire study year. In total, 47 high-sulfur protein genes were found to interact with ASE transcription factors and cofactors. The ASE transcription factors and cofactors were inhibited after melatonin implantation. We also constructed a regulatory network of tissue-specific genes and sulfur metabolism genes and found that melatonin possibly activates the sulfur metabolism process by regulating the genes related to cell energy metabolism and cell cycle in the skin. This can provide sufficient SAAs for cashmere wool growth. Our findings offer a new insight into wool growth regulation by sulfur metabolism genes and high-sulfur protein genes in cashmere goats.

## Data Availability Statement

The datasets analyzed for this study can be found in the Genome Sequence Archive in BIG Data Center, Beijing Institute of Genomics (BIG), Chinese Academy of Sciences, under accession numbers CRA004599 and CRA004598 that are publicly accessible at https://ngdc.cncb.ac.cn/gsa.

## Ethics Statement

The animal study was reviewed and approved by the Special Committee on Scientific Research and Academic Ethics of Inner Mongolia Agricultural University [Approval No. (2020)056].

## Author Contributions

YC: writing–original draft preparation and writing–reviewing and editing. WZ: supervision and project administration, and writing–reviewing and editing. YS, BL, LG, ZL, LZ, LD, and CJ: experimental sample and data analysis. CL: funding acquisition. YS: contributed as co-author. All authors contributed to the article and approved the submitted version.

## Conflict of Interest

BL was employed by the company Nei Mongol BioNew Technology Co. Ltd. The remaining authors declare that the research was conducted in the absence of any commercial or financial relationships that could be construed as a potential conflict of interest.

## Publisher’s Note

All claims expressed in this article are solely those of the authors and do not necessarily represent those of their affiliated organizations, or those of the publisher, the editors and the reviewers. Any product that may be evaluated in this article, or claim that may be made by its manufacturer, is not guaranteed or endorsed by the publisher.

## Footnotes

1
http://geneontology.org/

## Supplementary Material

Supplementary Figure 1 The average content of melatonin of cashmere goat in different months.Blue line is the treat group and red line is the control.

Supplementary Figure 2 KEGG pathway analysis of 856 genes.

Supplementary Figure 3 Expression pattern of 26 transcription cofactors and 22 transcription factors. D is the control group, M is the implantation group, and the number is the month.

Supplementary Figure 4 KEGG pathway of sulfur metabolism genes.

Supplementary Figure 5 **(A)** Global network of gene enrichment in the blood and skin samples of the control and treatment groups. **(B)** Pathways upregulated specifically in the treatment group skin and blood. **(C)** Pathways upregulated specifically in the control group skin and blood. **(D)** Upregulated specifically in the treatment and control group skin and blood. The red and blue node indicates that the pathway is up-regulated in the skin and blood, respectively, and the left and right halves of the node represent the treatment and control groups, respectively.

Supplementary Figure 6 Correlations between module eigengenes and sulfur metabolism genes. The numbers within the heat map represent correlations, and *P*-values (in parentheses; red, positively correlated, and blue, negatively correlated) for the module-trait associations.

Supplementary Figure 7 LEfSe highlights 23 tissue-specific consistently DEGs between the treatment group blood and skin.

## Abbreviations

ASE: allele specific expression
SAA: sulfur amino acids
PFs: primary follicles
SFs: secondary follicles
HQ: high quality
LQ: low quality
DEGs: differentially expressed genes
SNP: single nucleotide polymorphisms
WGCNA: weight gene co-expression network analysis
CDS: the coding sequences
LEfSe: linear discriminant analysis Effect Size.
