# Supplementary material for: Role of Sulfur Metabolism Gene and High-Sulfur Gene Expression in Wool Growth Regulation in the Cashmere Goat
Source: Front Genet. 2021 Aug 18;12:715526. doi: 10.3389/fgene.2021.715526 (PMC8416455; doi:10.3389/fgene.2021.715526)
Supplement: Supplementary Figure 1 — The average content of melatonin of cashmere goat in different months.Blue line is the treat group and red line is the control. [file Image_1.pdf]

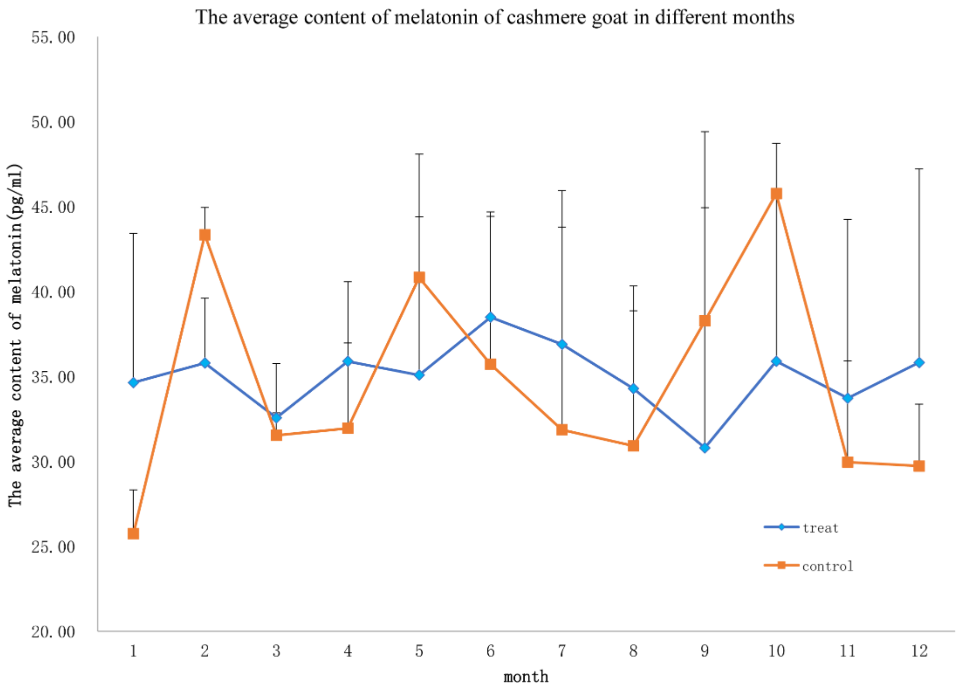


**Supplementary Figure1:** The average content of melatonin of cashmere goat in different months. Blue line is the treat group and red line is the control.
