# Supplementary figures and images for: Role of Sulfur Metabolism Gene and High-Sulfur Gene Expression in Wool Growth Regulation in the Cashmere Goat
Source: Front Genet. 2021 Aug 18;12:715526. doi: 10.3389/fgene.2021.715526 (PMC8416455; doi:10.3389/fgene.2021.715526)

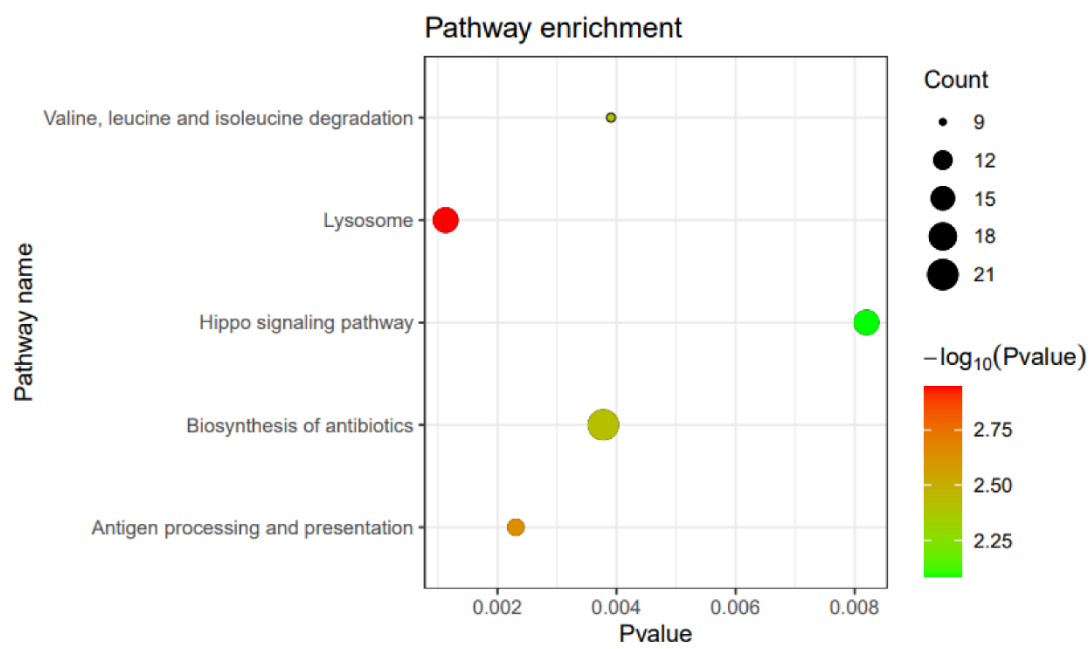

**Supplementary Figure2:** KEGG pathway analysis of 856 genes.

Supplement: Supplementary Figure 2 — KEGG pathway analysis of 856 genes. [file Image_2.pdf]

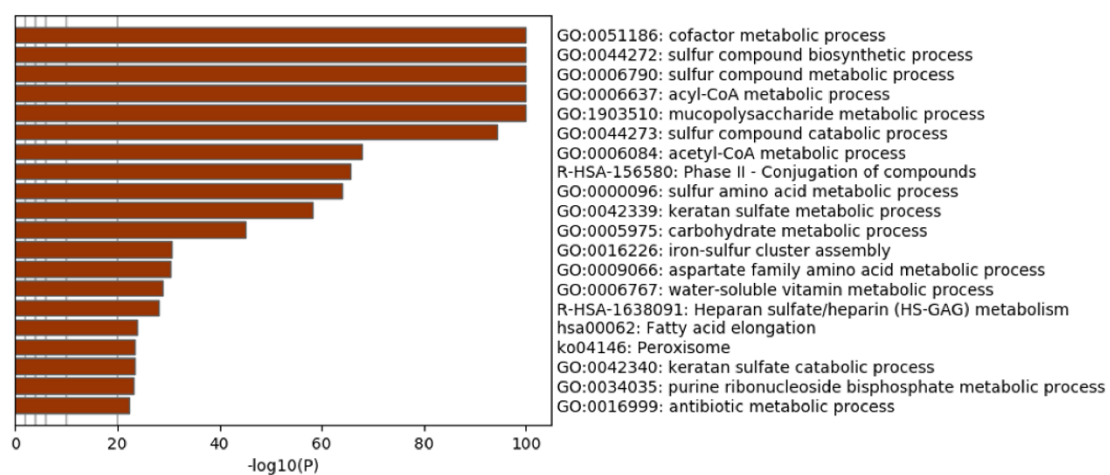

**Supplementary Figure4:** KEGG pathway of sulfur metabolism genes.

Supplement: Supplementary Figure 4 — KEGG pathway of sulfur metabolism genes. [file Image_4.pdf]
