# Supplementary material for: Role of Sulfur Metabolism Gene and High-Sulfur Gene Expression in Wool Growth Regulation in the Cashmere Goat
Source: Front Genet. 2021 Aug 18;12:715526. doi: 10.3389/fgene.2021.715526 (PMC8416455; doi:10.3389/fgene.2021.715526)
Supplement: Supplementary Figure 3 — Expression pattern of 26 transcription cofactors and 22 transcription factors. D is the control group, M is the implantation group, and the number is the month. [file Image_3.pdf]

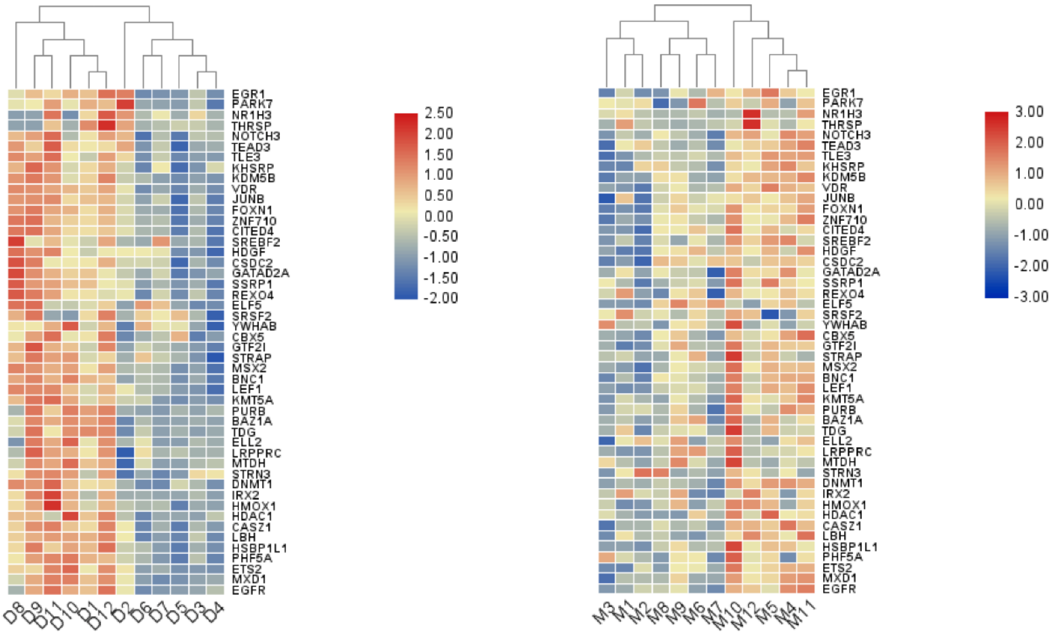

A B

Supplementary Figure3: Expression pattern of 26 transcription cofactors and 22 transcription factors. D is the control group, M is the implantation group, and the number is the month
