# Supplementary material for: Role of Sulfur Metabolism Gene and High-Sulfur Gene Expression in Wool Growth Regulation in the Cashmere Goat
Source: Front Genet. 2021 Aug 18;12:715526. doi: 10.3389/fgene.2021.715526 (PMC8416455; doi:10.3389/fgene.2021.715526)
Supplement: Supplementary Figure 6 — Correlations between module eigengenes and sulfur metabolism genes. The numbers within the heat map represent correlations, and P-values (in parentheses; red, positively correlated, and blue, negatively correlated) for the module-trait associations. [file Image_6.pdf]

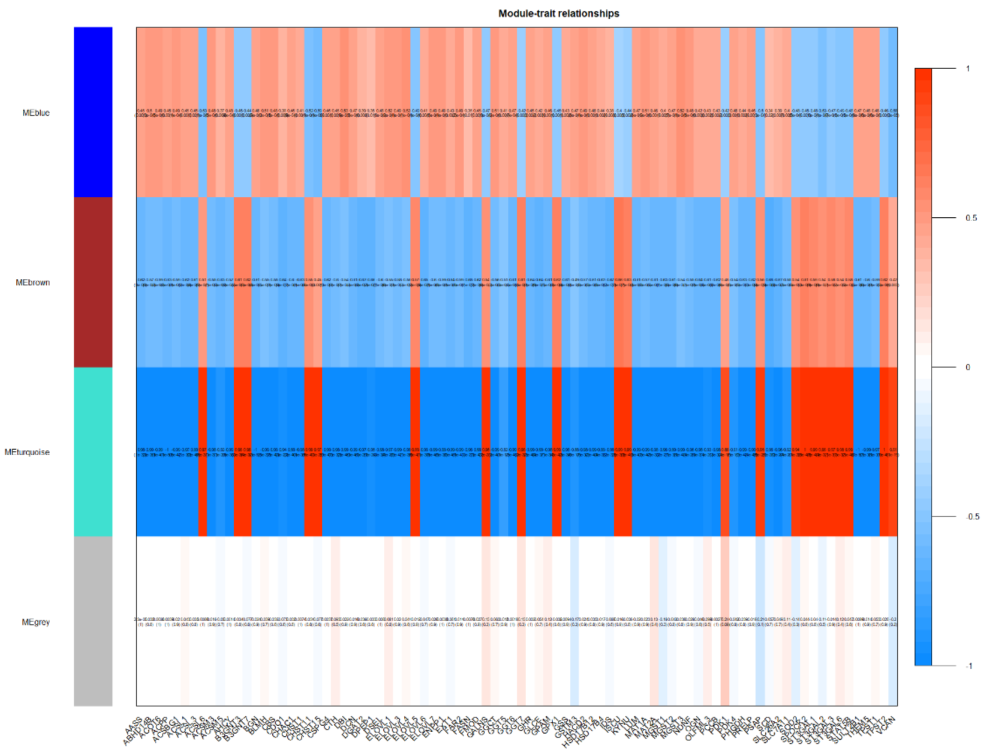

Supplementary Figure6: Correlations between module eigengenes and sulfur metabolism genes. The numbers within the heat map represent correlations, and *P* values in parentheses; red, positively correlated, and blue, negatively correlated for the module-trait associations.
