# Supplementary material for: Role of Sulfur Metabolism Gene and High-Sulfur Gene Expression in Wool Growth Regulation in the Cashmere Goat
Source: Front Genet. 2021 Aug 18;12:715526. doi: 10.3389/fgene.2021.715526 (PMC8416455; doi:10.3389/fgene.2021.715526)
Supplement: Supplementary Figure 7 — LEfSe highlights 23 tissue-specific consistently DEGs between the treatment group blood and skin. [file Image_7.pdf]

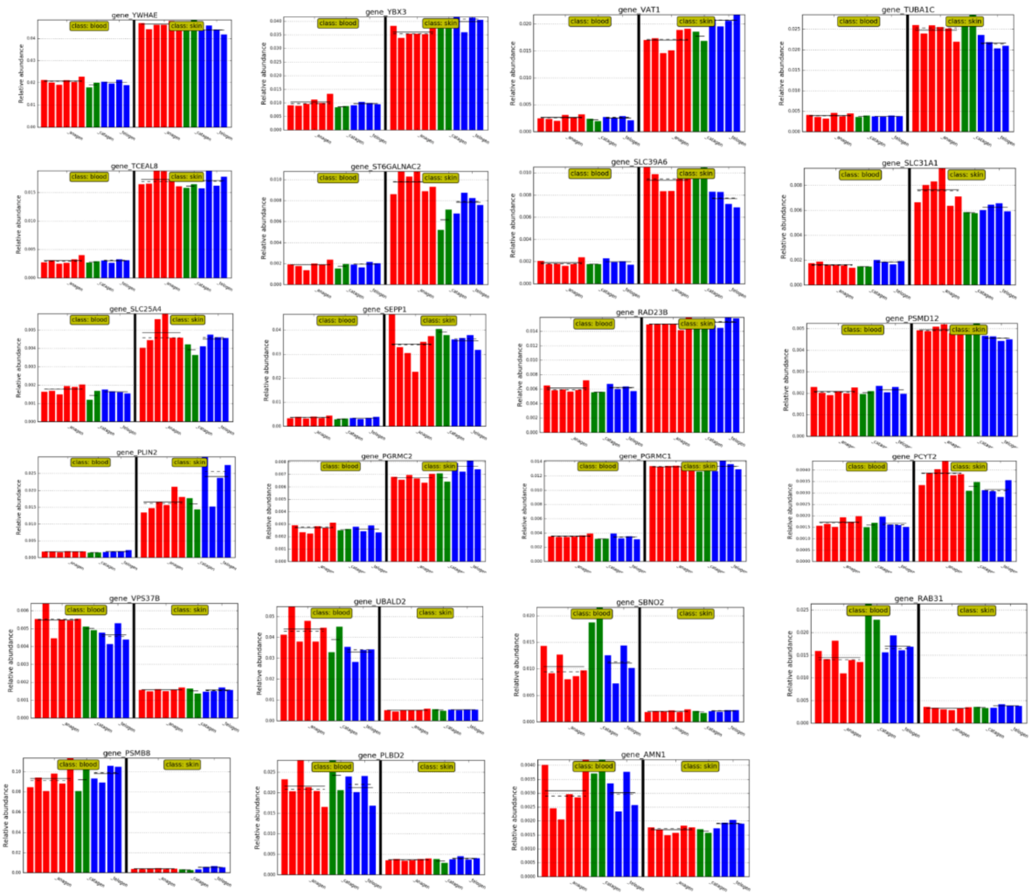

Supplementary Figure7: LEfSe highlights 23 tissue-specific consistently DEGs between the treatment group blood and skin.
