# Supplementary material for: Role of Sulfur Metabolism Gene and High-Sulfur Gene Expression in Wool Growth Regulation in the Cashmere Goat
Source: Front Genet. 2021 Aug 18;12:715526. doi: 10.3389/fgene.2021.715526 (PMC8416455; doi:10.3389/fgene.2021.715526)
Supplement: Supplementary Figure 5 — (A) Global network of gene enrichment in the blood and skin samples of the control and treatment groups. (B) Pathways upregulated specifically in the treatment group skin and blood. (C) Pathways upregulated specifically in the control group skin and blood. (D) Upregulated specifically in the treatment and control group skin and blood. The red and blue node indicates that the pathway is up-regulated in the skin and blood, respectively, and the left and right halves of the node represent the treatment and control groups, respectively. [file Image_5.pdf]

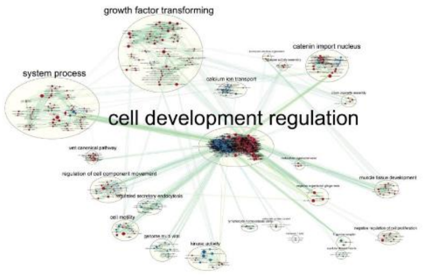

A

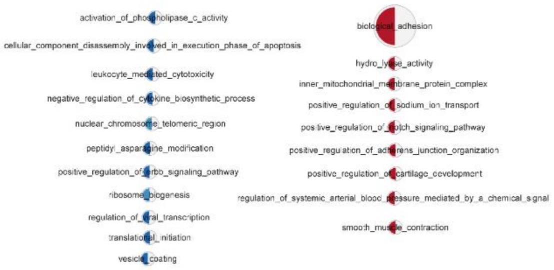

B

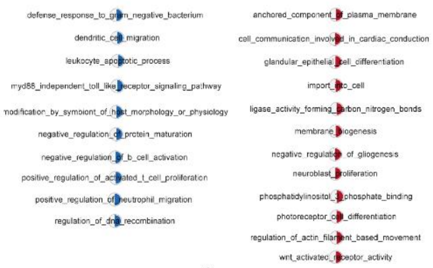

C

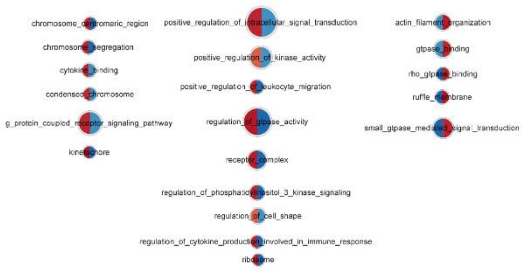

D

Supplementary Figure5: A**:** Global network of gene enrichment in the blood and skin samples of the control and treatment groups. B: Pathways upregulated specifically in the treatment group skin and blood. C: Pathways upregulated specifically in the control group skin and blood. D: Upregulated specifically in the treatment and control group skin and blood. The red and blue node indicates that the pathway is up-regulated in the skin and blood, respectively, and the left and right halves of the node represent the treatment and control groups, respectively.
